# Data Augmentation: Using Channel-Level Recombination to Improve Classification Performance for Motor Imagery EEG

**DOI:** 10.3389/fnhum.2021.645952

**Published:** 2021-03-11

**Authors:** Yu Pei, Zhiguo Luo, Ye Yan, Huijiong Yan, Jing Jiang, Weiguo Li, Liang Xie, Erwei Yin

**Affiliations:** ^1^School of Software, Beihang University, Beijing, China; ^2^Tianjin Artificial Intelligence Innovation Center (TAIIC), Tianjin, China; ^3^Unmanned Systems Research Center, National Innovation Institute of Defense Technology, Academy of Military Sciences China, Beijing, China; ^4^National Key Laboratory of Human Factors Engineering, China Astronaut Research and Training Center, Beijing, China

**Keywords:** brain-computer interface, electroencephalogram, motor imagery, deep learning, inter-subject transfer learning, pre-training, data augmentation

## Abstract

The quality and quantity of training data are crucial to the performance of a deep-learning-based brain-computer interface (BCI) system. However, it is not practical to record EEG data over several long calibration sessions. A promising time- and cost-efficient solution is artificial data generation or data augmentation (DA). Here, we proposed a DA method for the motor imagery (MI) EEG signal called brain-area-recombination (BAR). For the BAR, each sample was first separated into two ones (named half-sample) by left/right brain channels, and the artificial samples were generated by recombining the half-samples. We then designed two schemas (intra- and adaptive-subject schema) corresponding to the single- and multi-subject scenarios. Extensive experiments using the classifier of EEGnet were conducted on two public datasets under various training set sizes. In both schemas, the BAR method can make the EEGnet have a better performance of classification (*p* < 0.01). To make a comparative investigation, we selected two common DA methods (noise-added and flipping), and the BAR method beat them (*p* < 0.05). Further, using the proposed BAR for augmentation, EEGnet achieved up to 8.3% improvement than a typical decoding algorithm CSP-SVM (*p* < 0.01), note that both the models were trained on the augmented dataset. This study shows that BAR usage can significantly improve the classification ability of deep learning to MI-EEG signals. To a certain extent, it may promote the development of deep learning technology in the field of BCI.

## 1. Introduction

The brain-computer interface (BCI) is a communication control system directly established between the brain and external devices (computers or other electronic devices), using signals generated during brain activity (Wolpaw et al., 2000). Instead of relying on the muscles and organs, the system directly builds communication between the brain and the machine. Electroencephalogram (EEG) is one of the most common signals used for building a BCI system because of its cost-effectiveness, non-invasive implementation, and portability. BCIs have shown potentials in applying various fields such as communication, control, and rehabilitation (Abdulkader et al., 2015).

Recent years have witnessed intense researches into different types of BCI systems. According to the signal acquisition method, BCI technology can be divided into three types: non-implantable system, semi-implantable system, an implantable system (Wolpaw et al., 2000). Non-implantable BCI systems mainly use EEG to recognize human's intention. According to the signal generation mechanism, BCI systems can be divided into induced BCI systems and spontaneous BCI systems. The induced BCI systems are: steady-state visual evoked potentials (SSVEP) (Friman et al., 2007; Ko et al., 2020), slow cortical potentials (Beuchat et al., 2013), and the P300 (Yin et al., 2016; Yu et al., 2017; Chikara and Ko, 2019), and the spontaneous BCI systems are: motor imagery (MI) (Choi and Cichocki, 2008; Belkacem et al., 2018; Chen et al., 2019; Wang et al., 2020).

The motor imagery (MI) BCI system's framework is based on the fact that the brain's activity in a specific area will be changed when the patients (or subjects) imagine moving any part of their bodies. For example, when a person imagines moving his/her right arm, there is a desynchronization of neural activity in the primary motor cortex on the left side of the brain. This desynchronization is called event-related desynchronization (ERD), which can be observed in the EEG signals transitioning from resting-state energy level to a lower energy level. The spatial location, temporal onset, amount of decrease, and ERD's stability are all subject-dependent factors (McFarland et al., 2000; Lotze and Halsband, 2006), bringing challenges for detecting changes in MI's neural activity.

In recent years, based on the considerable amount of data and sophisticated model structure, deep learning has been proved its strong learning ability to classify linguistic features, images, and sounds (Zhong et al., 2015; Song et al., 2017; Alom et al., 2018; Cooney et al., 2019). However, it is difficult to collect sufficient data in practice due to the limited available subjects, experimental time, and operation complexity in BCI. This problem is pronounced in MI-based BCI. The performance of deep neural networks (DNNs) is susceptible to the number of samples. A small scale dataset often leads to poor generalization during model training, reducing the decoding accuracy (LeCun et al., 2015).

A feasible approach to improve deep networks' performance and to avoid the overfitting caused by lack of training data is data augmentation (DA) methods (Salamon and Bello, 2017). These methods augment training data by artificially generating new samples based on existing data (Roy et al., 2019). Yin and Zhang (2017) added Gaussian white noise to the EEG feature vector to improve their deep learning model's accuracy on the classification task of Mental Workload (MW). Sakai et al. (2017) shifted EEG trials in time axis and amplified the amplitude to generate artificial EEG signals for augmentation. The results showed that their augmentation method improved the classification performance when the training set's size was 20, but this method has no significant effect on the more extensive training set. In another work, artificial EEG trials were generated by segmentation and recombination in time and frequency domains (Lotte, 2015), and the results were more convincing. Other studies have used more advanced techniques such as variational auto-encoders (VAE) (Aznan et al., 2019) and generative adversarial networks (GANs) (Goodfellow et al., 2014). However, tens of thousands of parameters in these methods need to be trained using the original data, which creates a certain degree of demand for the original data scale. It is a conflict with our goal of data augmentation on a tiny training set. Besides, the huge consumption of computing resources and the difficulty of being reproduced are also their shortcomings, although they have achieved a certain degree of success in some aspects (Karras et al., 2017; Kodali et al., 2017).

We proposed a new motor imagery EEG DA method, called Brain Area Recombination (BAR), which first decomposes the training dataset from the left and right brains and reassembles them into a new training dataset. Pre-training on the datasets of other subjects is also a common way to solve the insufficient training of deep neural networks (Fahimi et al., 2020). There are two types of pre-training, one is to use the source subjects' data in the same dataset as the pre-training training dataset, and the other is to use another dataset as the pre-training training dataset (Xu et al., 2020). The first type of pre-training is used in our study. Fortunately, experiments show that our method can be well-embedded with the pre-training framework to improve the deep learning network's classification performance.

Compared with the methods above, the proposed BAR has the following advantages:

1. Low computational complexity;
2. High and fixed expansion ratio;
3. Great quality of new artificial samples.

This paper's remainder is organized as follows: section 2 introduced the public dataset used in the study. Section 3 proposed a preliminary experiment and our method's hypothesis in detail and then introduced the method's mathematical definition. We used two common schemas to evaluate our method and implemented the other two common DA methods as a comparison. Experiment results were showed in section 4, which demonstrated that the proposed BAR had achieved significant results. Section 5 presented the discussion. Section 6 concluded the study.

## 2. Materials and Methods

### 2.1. Materials

**Figure 1:**
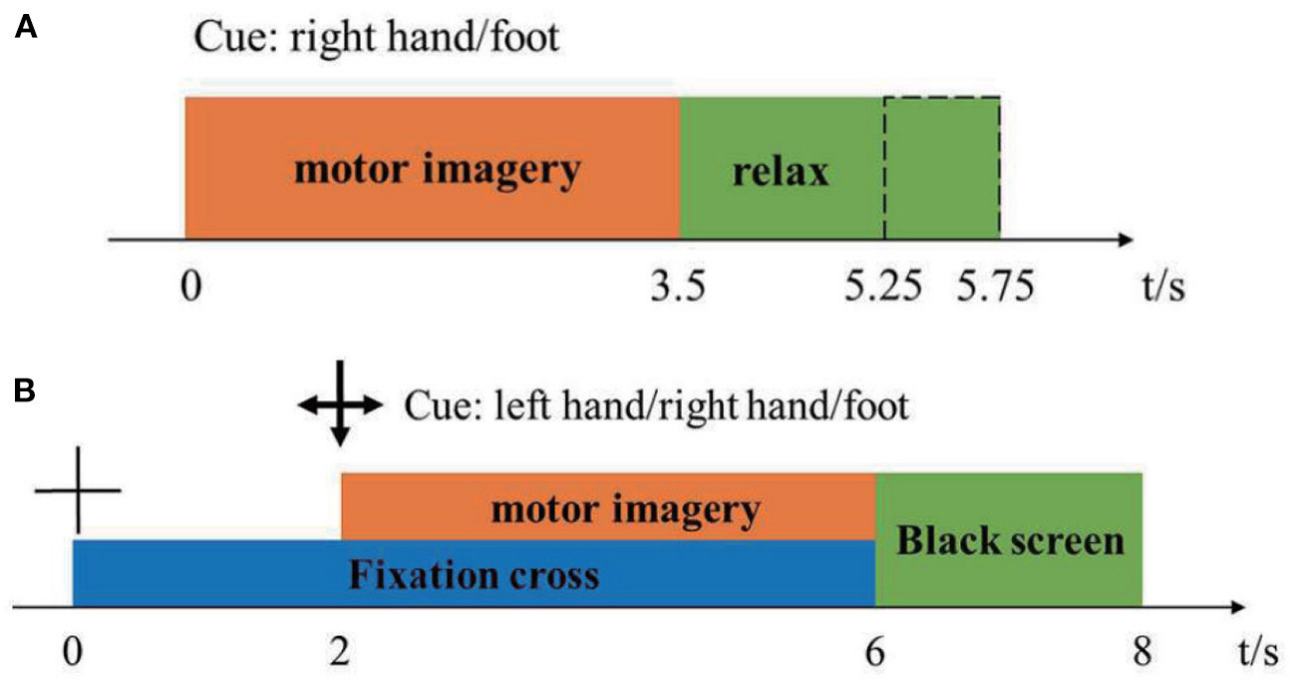
Timeline of one trial in the dataset 1 **(A)** and dataset 2 **(B)**.

Dataset 1: The first dataset was from BCI-Competition-III-IVa and was collected in a cue-based setting. Only cues for the classes “right” and “foot” are provided. This dataset was recorded from five healthy subjects (aa, al, av, aw, ay) at 100 Hz. The subjects sat in a comfortable chair with arms resting on armrests. The timeline of the dataset was shown in Figure 1A. The raw data were continuous signals of 118 EEG channels and markers that indicate the time points of 280 cues. Each sample was segmented from [0, 2.5] s by marks, then passed a band-pass filter (5-order Butterworth digital filter with cut-off frequencies at [8, 30] Hz) to remove muscle artifacts, line-noise contamination, and DC drifts. Under the condition that the positive sample and the negative sample were balanced, 100 samples were randomly selected as the training pool. The remaining samples were used as the test samples. The details of the competition, including ethical approval, and the raw data can be download from http://www.bbci.de/competition/iii/.

Dataset 2: The second dataset was from BCI-Competition-IV-1. The dataset was recorded from seven healthy subjects (a, b, c, d, e, f, g), including four healthy individuals (named “a,” “b,” “f,” “g”) and three artificially generated “participants” (named “c,” “d,” “e”). 59-channel EEG signals were recorded at 100 Hz. Two motor imagery classes were selected for each subject from the three classes: left hand, right hand, and foot. The timeline of the dataset was shown in Figure 1B. There were two subjects (a, f) whose motor imaging tasks were different from the others, so they were eliminated. Here we only used the calibration data because of the complete marker information. Each sample was segmented from [0, 2.5] s by marks, then passed a band-pass filter (5-order Butterworth digital filter with cut-off frequencies at [8, 30] Hz) to remove muscle artifacts, line-noise contamination, and DC drifts. After preprocessing, we obtained 200 samples for each subject. We randomly selected 100 samples as a training pool and the rest as test samples, like dataset 1. The details of the competition, including ethical approval, and the raw data can be download from http://www.bbci.de/competition/iv/.

### 2.2. Methods

#### 2.2.1. Core Assumption

Consider that we select two samples from the original samples randomly, and take out the left brain part of the first sample and the right brain part of the second sample, and recombine these two parts together to form an artificial sample. This artificial sample is still a normal EEG sample (1).

(1) $$\begin{array}{l} \text{I}\text{f}\;x_{i},x_{j}\sim P_{MI-EEG},\;\text{Then}\;\hat{x}=\left[\begin{array}{c} x_{i}^{\left(R\right)}\\ x_{j}^{\left(L\right)} \end{array}\right]\sim P_{MI-EEG} \end{array}$$

where $x_{i},x_{j}\in {\mathbb{R}}^{C\times T}$, *C* is the number of electrodes, *T* is the sample-points, *P*_*MI*−*EEG*_ is the distribution of MI-EEG data. $x^{\left(R\right)},x^{\left(L\right)}\in {\mathbb{R}}^{\frac{C}{2}\times T}$ represent samples containing only the right brain channels and the left brain channels, respectively.

#### 2.2.2. Brain Area Recombination

**Figure 2:**
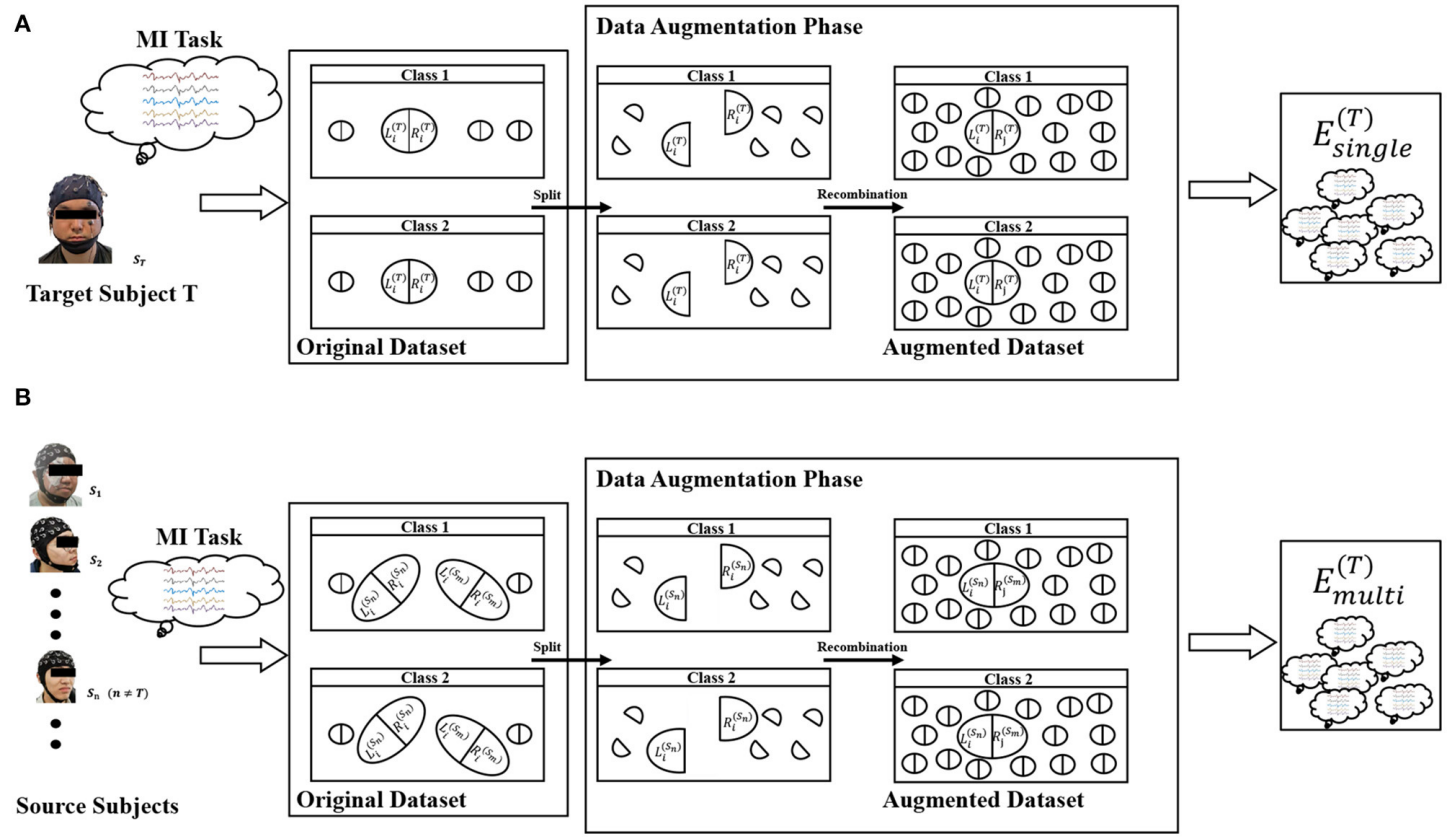
Illustration of the Equation (2) **(A)** and Equation (3) **(B)**.

Based on the assumption described in (1), we propose two similar DA methods for single-subject and multi-subject scenes for EEG of motor imagination. The whole framework of our proposed method was shown in Figure 2.

For the single-subject scene, the data augmentation method is:

(2) $$\begin{array}{l} E_{single}^{\left(s\right)}=\overset{N_{c}}{\underset{c=1}{⋃}}\left\{x^{\left(R\right)}|x^{\left(R\right)}\in D_{s}^{\left(R\right)},y\left(x^{\left(R\right)}\right)=c\right\}\\ \begin{array}{l} \;\times \left\{x^{\left(L\right)}|x^{\left(L\right)}\in D_{s}^{\left(L\right)},y\left(x^{\left(L\right)}\right)=c\right\} \end{array} \end{array}$$

For the multi-subject scene, the data augmentation method is:

(3) $$\begin{array}{l} E_{multi}^{\left(s\right)}=\overset{N_{s}}{\underset{i,j\neq s}{⋃}}\overset{N_{c}}{\underset{c=1}{⋃}}\left\{x^{\left(R\right)}|x^{\left(R\right)}\in D_{i}^{\left(R\right)},y\left(x^{\left(R\right)}\right)=c\right\}\\ \begin{array}{l} \;\times \left\{x^{\left(L\right)}|x^{\left(L\right)}\in D_{j}^{\left(L\right)},y\left(x^{\left(L\right)}\right)=c\right\} \end{array} \end{array}$$

where *N*_*s*_ and *N*_*c*_ represent the number of subjects and the number of classification tasks, respectively. *y*(·) is a mapping to get the label. *s* is the index of the subject. $D_{i}^{\left(R\right)}$ and $D_{i}^{\left(L\right)}$ represent the training dataset from the right brain and the left brain for the i-th subject, respectively. “ × ” was defined as the cartesian-like product operator. For example, there are two matrix sets *A* = {*a*_1_, *a*_2_}, *B* = {*b*_1_, *b*_2_} and their product $C=A\times B=\{\left[\begin{array}{c} a_{1}\\ b_{1} \end{array}\right],\left[\begin{array}{c} a_{1}\\ b_{2} \end{array}\right],\left[\begin{array}{c} a_{2}\\ b_{1} \end{array}\right],\left[\begin{array}{c} a_{2}\\ b_{2} \end{array}\right]\}.$ Note that $E_{multi}^{\left(s\right)}$ does not contain any sample from the s-th subject. In other words, it is a cross-subject training dataset. Figure 2A represents the meaning of Equation (2) and Figure 2B represents the meaning of Equation (3). The zero midline electrodes are Fpz, Fz, FCz, Cz, CPz, Pz, POz, and Oz in dataset 1. We alternately divide them into two sets in turn. Specifically, Fpz, FCz, CPz, and Pz are selected to be the brain's left part, and Fz, Cz, Pz, Oz were are selected to be the brain's right part. In dataset 2, the zero midline electrodes are Fz, FCz, Cz, CPz, and Pz. Fz, Cz, and Pz are selected to be the brain's left part. FCz and CPz were are selected to be the brain's right part.

#### 2.2.3. Noise-Added and Flipping

The problem we want to solve is data augmentation on a tiny training dataset. Methods like GANs and VAEs having massive parameters to be learned are not suitable for this situation. Moreover, considering that the no-parameter (or few parameters) methods will often achieve better results for this situation, we selected noise-added and flipping methods for comparative investigation (Lashgari et al., 2020). Concerning the noise-added method, a Gaussian noise matrix with SNR (signal to noise ratio) of 5 is calculated, and then this noise matrix is added to the original sample. The rule of the flipping DA method is to reverse each real sample in the time axis. Because the noise-added DA method does not have a fixed expansion ratio constant, we have implemented two versions of the noise-added DA method for a more objective comparison. One implementation (version 1) makes the noise-added DA method have the same expansion ratio as the flipping DA method. The other implementation (version 2) makes the noise-added DA method and the proposed BAR DA method have the same expansion ratio constant.

#### 2.2.4. The EEGnet

We use the end-to-end deep learning model, named EEGnet (Lawhern et al., 2018). The EEGnet takes the EEG segments as the input, passes them through three convolution layers for feature extraction, and uses a fully connected layer to classify. The first layer is a temporal convolution to learn frequency filters. The second layer is a depthwise convolution layer. This layer connects to each feature map individually and learns frequency-specific spatial filters. The third layer is a separable convolution layer. The separable convolution is a combination of depthwise convolution, which learns a temporal summary for each feature map individually, followed by a pointwise convolution, which learns how to mix the feature maps optimally. All feature maps are flattened and are fed into a fully connected layer. Full details about the network architecture can be found in the open-source project: https://github.com/vlawhern/arl-eegmodels. In this study, we use the default hyperparameters provided by the open-source project.

#### 2.2.5. The Common Spatial Patterns Extraction

Due to the strong spatial distribution characteristics of motor imagery EEG signals, a feature extraction method called Common Spatial Patterns (CSP) is designed (Koles et al., 1990). The CSP aims to construct spatial filters which can maximize the variance of band-pass filtered EEG signals from one class and minimize the variance of EEG signals from the other class (Lotte et al., 2018). Formally, CSP uses the spatial filters *w* to assign a weight to each EEG sample channel. The *w* can be calculated by extremizing the following function:

(4) $$\begin{array}{l} J\left(w\right)=\frac{w^{\prime }X_{1}^{\prime }X_{1}w}{w^{\prime }X_{2}^{\prime }X_{2}w}=\frac{w^{\prime }C_{1}w}{w^{\prime }C_{2}w} \end{array}$$

By maximizing Equation (4), we can calculate the spatial filter focusing on class 1. Indeed, *J*(*k* × *w*) = *J*(*w*), with *k* a real constant, means that the rescaling of the *w* is arbitrary. To calculate the only maximizer, we need a condition that $w^{\prime }C_{2}w=1$. Using the Lagrange multiplier method, the constrained optimization problem is equivalent to maximizing the following function:

(5) $$\begin{array}{l} L\left(\lambda ,w\right)=w^{\prime }C_{1}w-\lambda \left(w^{\prime }C_{2}w-1\right) \end{array}$$

The filters *w* maximizing *L* can be calculated by setting the derivative of *L* concerning *w* to 0:

(6) $$\begin{array}{l} \frac{\partial L}{\partial w}=2w^{\prime }C_{1}-2\lambda w^{\prime }C_{2}=0\\ \;\Leftrightarrow C_{1}w=\lambda C_{2}w\\ \;\Leftrightarrow C_{2}^{-1}C_{1}w=\lambda w \end{array}$$

We obtain an eigenvalue problem in Equation (6). Therefore, the spatial filters maximizing Equation (4) are the eigenvectors of $M=C_{2}^{-1}C_{1}$ which correspond to its largest eigenvalue. Empirically, we select the eigenvectors corresponding to the top *k*(*k* = 3) eigenvalues and concatenate them into a matrix $W_{N_{c}\times k}^{\left(1\right)}$. This matrix can capture the three components that most relate to class 1. For the class 2, we can swap the numerator and denominator in the Equation (4) and repeat the above process. Finally, we concatenate the two matrices together to obtain the CSP-feature extraction matrix $W=\left[W_{N_{c}\times k}^{\left(1\right)},W_{N_{c}\times k}^{\left(2\right)}\right]$.

## 3. Results

**Figure 3:**
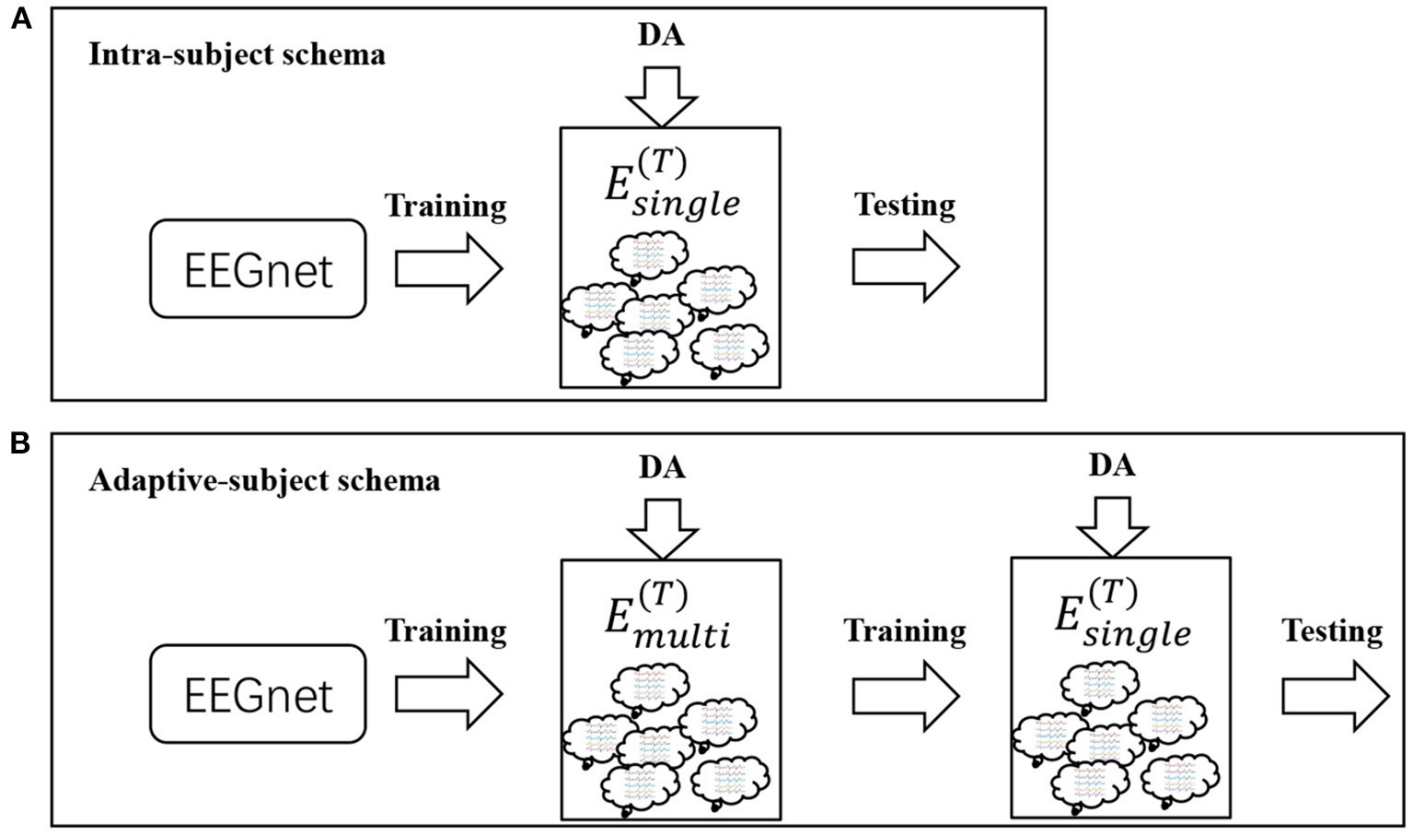
Workflows for IS-schema and AS-schema. Panel **(A)** is the workflow of intra-subject-schema and panel **(B)** is the workflow of adaptive-subject-schema.

We designed two schemas to evaluate these DA methods. Both schemas used the same deep learning model called EEGnet (Lawhern et al., 2018). The setting of the dataset was different between both schemas. In the first schema, referred to as intra-subject(IS)-schema, each subject's EEGnet was only trained on the subject's own dataset. In the second schema, referred to as adaptive-subject(AS)-schema, each subject's EEGnet should be trained in two stages. In the first stage, named the pre-training stage, the EEGnet should be trained on other subjects' datasets. In the second stage, named the adaptive-training stage, the EEGnet should be trained on the target subject's dataset. Figure 3 showed the two schemas' workflow. We only used one DA method in each experiment instead of experimenting with multiple DA methods' additive effects. Two DA methods (noise-added and flipping) were implemented as reference methods (Lashgari et al., 2020). The flipping DA method flipped each sample along the time axis to generate a new sample. So this DA method can only get a double-sized dataset. The noise-added DA method added noise to each sample to generate new samples. Since the noise-added DA method had no fixed expansion factor, we implemented two versions with different expansion factors for the more objective comparison experiments. We repeated 10 times of experiments in each training set size for a specific subject.

### 3.1. IS-Schema Performance

**Figure 4:**
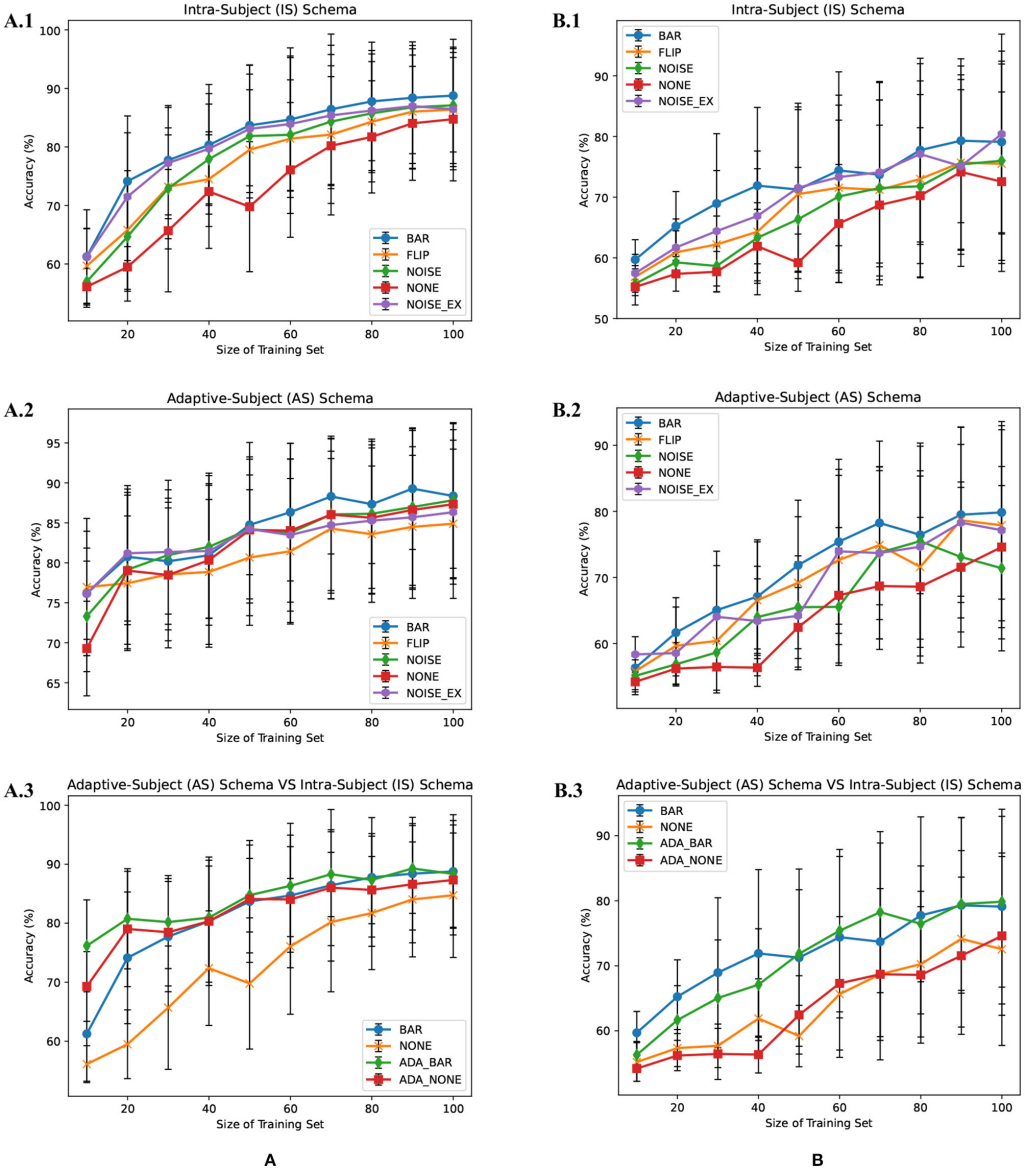
Average subjects performance on different size of training dataset in the IS-schema and AS-schema from dataset 1 **(A)** and dataset 2 **(B)** was plotted. The red line with “NONE” in **(A.1,A.2,B.1,B.2)** indicates the case that the training set was not augmentated. The lines with “NOISE” and “NOISE_EX” represent version 1 of noise-added method and version 2 of the noise-added method, respectively. The green line and red line with the prefix “ADA_” in **(A.3,B.3)** represent AS-schema's accuracies. The blue line and yellow line in **(A.3,B.3)** represent IS-schema's accuracies.

**Figure 5:**
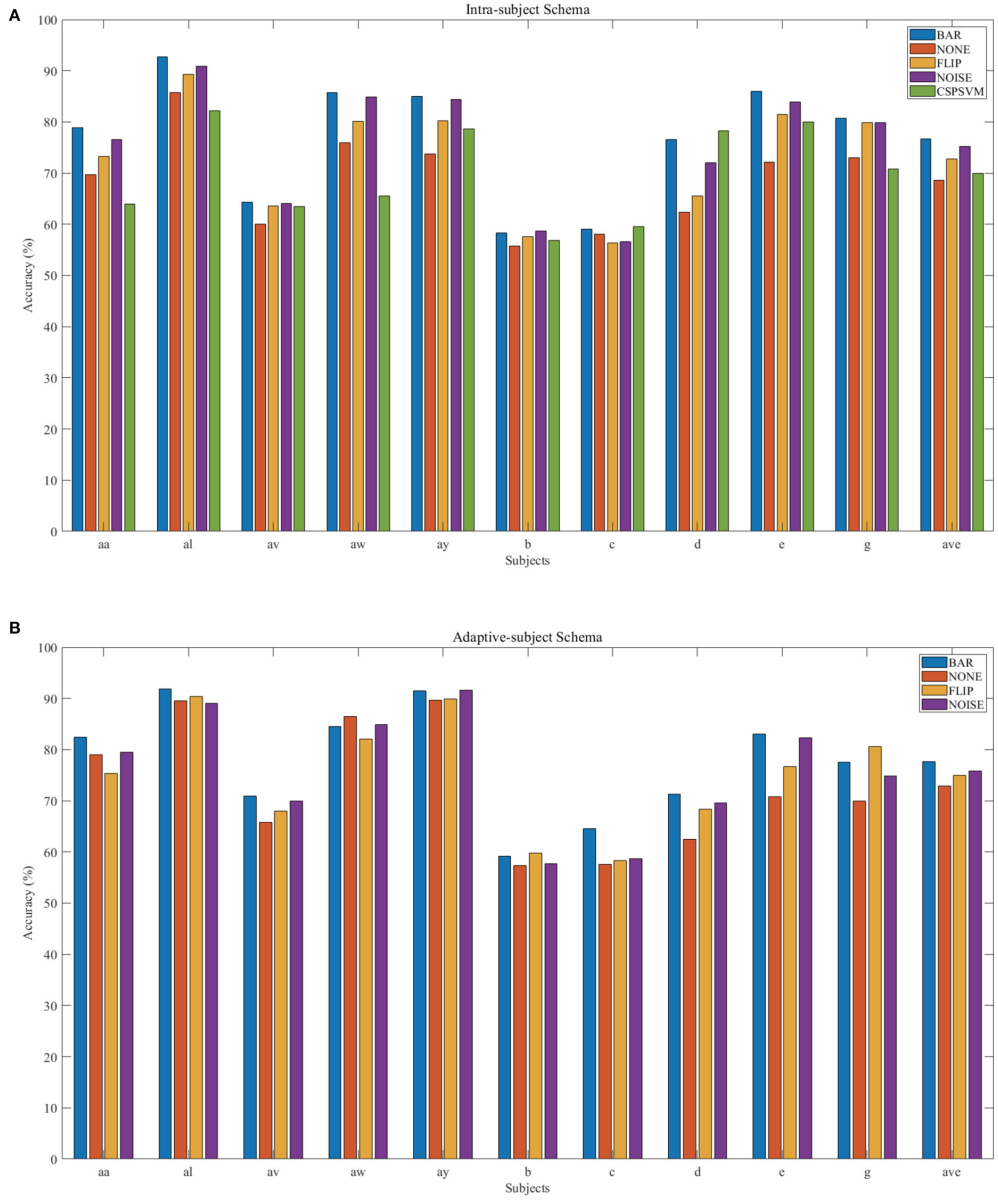
Individual result in IS-schema **(A)** and AS-schema **(B)**. Note that the green bar (“CSPSVM”) in **(A)** is the traditional classifier's result using the BAR method. The bar named “NOISE” represents the version 2 of noise-added method.

There were multiple subjects in each dataset. For the *s*_*th*_ subject, we first randomly selected some samples to construct the original-training dataset *D*_*s*_. The augmentation methods were applied to the *D*_*s*_ in turn to construct the corresponding augmented training datasets. The EEGnet was trained on these training datasets separately and was tested on the testing dataset. To test the proposed BAR's sensitivity to the training dataset's size, we conducted extensive experiments on different training dataset sizes. For a specific subject, we repeated the experiment 10 times on a specific training set size from 10 to 100 and then took the averaged accuracy as the final one. The results of those experiments were plotted in Figures 4A.1,B.1, 5A. Figures 4A.1,B.1 shown the performance of each DA method under different training set sizes. This performance was the average result of all subjects. For dataset 1, as the size of the training set increases, all DA methods' performance is improving, but our BAR method is always ahead of other methods by about 3%, except for the case where the training set size is 10. Figure 5A shown the performance of each DA method on different subjects. These accuracies come from the averaged accuracy of all experiments. For a specific subject, we run many experiments on different training set sizes from 10 to 100, and all accuracies are averaged to be the final one. A paired-sample *t*-test was used to measure the significance of our proposed BAR, and the results were shown in Tables 1, **3**. The test results were *p* < 0.005 between the referenced methods and the proposed method.

**Table 1:** Paired-sample *t*-test result on the test accuracy in Figures 4A.1,B.1.

**Dataset 1**				
	**FLIP**	**NOISE**	**NONE**	**NOISE_EX**
BAR	1 × 10^−4^	2 × 10^−3^	1 × 10^−5^	2 × 10^−3^
**Dataset 2**				
	**FLIP**	**NOISE**	**NONE**	**NOISE_EX**
BAR	1 × 10^−4^	8 × 10^−6^	6 × 10^−7^	2.6 × 10^−3^

### 3.2. AS-Schema Performance

In the AS-schema: For each subject, we selected the same samples randomly from other subjects to construct the training dataset. In this schema, two training sets were needed, and the EEGnet was trained on them as Figure 3 shows. The first dataset was a cross-subject dataset which is constructed by (3) for the *s*_*th*_ subject. The second dataset was constructed by (2), which was the same as the training dataset in the intra-subject schema. For the flipping method, we first mix the source subjects' data and then flip each sample in this mixed dataset to obtain a new artificial sample. For the noise-added method, we have two versions of strategies. Version 1: We first mix the source subjects' data and then add gaussian noise to each sample in this mixed dataset to obtain a new artificial sample. Version 2: We randomly select an original sample with replacement from the source subjects' mixed data and add Gaussian noise to it to obtain a new artificial sample. Repeat the process until the original samples, and the artificial samples are equals to the augmented dataset by the BAR. To investigate our proposed BAR's performance in different sizes of selected samples for each subject, we run the adaptive-subject experiment many times in each size of selected samples. The result was demonstrated in Figures 4A.2,B.2, 5B. Figures 4A.2,B.2 shown the performance of each DA method under different training set sizes. This performance was the average result of all subjects. For dataset 1 and dataset 2, as the training set size increases, all DA methods' performance increases, but our BAR method has always been ahead of other methods except for a few cases. Figure 5B shown the performance of each DA method on different subjects. These accuracies come from the averaged accuracy of all experiments. For a specific subject, we run a lot of experiments on different training set sizes from 10 to 100, and all accuracies are averaged to be the final one. A Paired-sample *t*-test was used to measure our proposed BAR's significance, and the result was shown in Tables 2, 3. The test results were *p* < 0.05 between the referenced methods and the proposed method.

**Table 2:** Paired-sample *t*-test result on the test accuracy in Figures 4A.2,B.2.

**Dataset 1**				
	**FLIP**	**NOISE**	**NONE**	**NOISE_EX**
BAR	2 × 10^−4^	2 × 10^−3^	4 × 10^−3^	5 × 10^−2^
**Dataset 2**				
	**FLIP**	**NOISE**	**NONE**	**NOISE_EX**
BAR	9 × 10^−5^	3 × 10^−4^	6 × 10^−7^	1.2 × 10^−3^

**Table 3:** Paired-sample *t*-test result on the test accuracy in Figure 5.

**IS-schema**				
	**FLIP**	**NOISE**	**NONE**	**CSPSVM**
BAR	3 × 10^−3^	7.2 × 10^−3^	3 × 10^−4^	1.5 × 10^−2^
**AS-schema**				
	**FLIP**	**NOISE**		**NONE**
BAR	2.3 × 10^−2^	9.9 × 10^−4^		5.2 × 10^−3^

### 3.3. EEGnet vs. CSP-SVM

**Figure 6:**
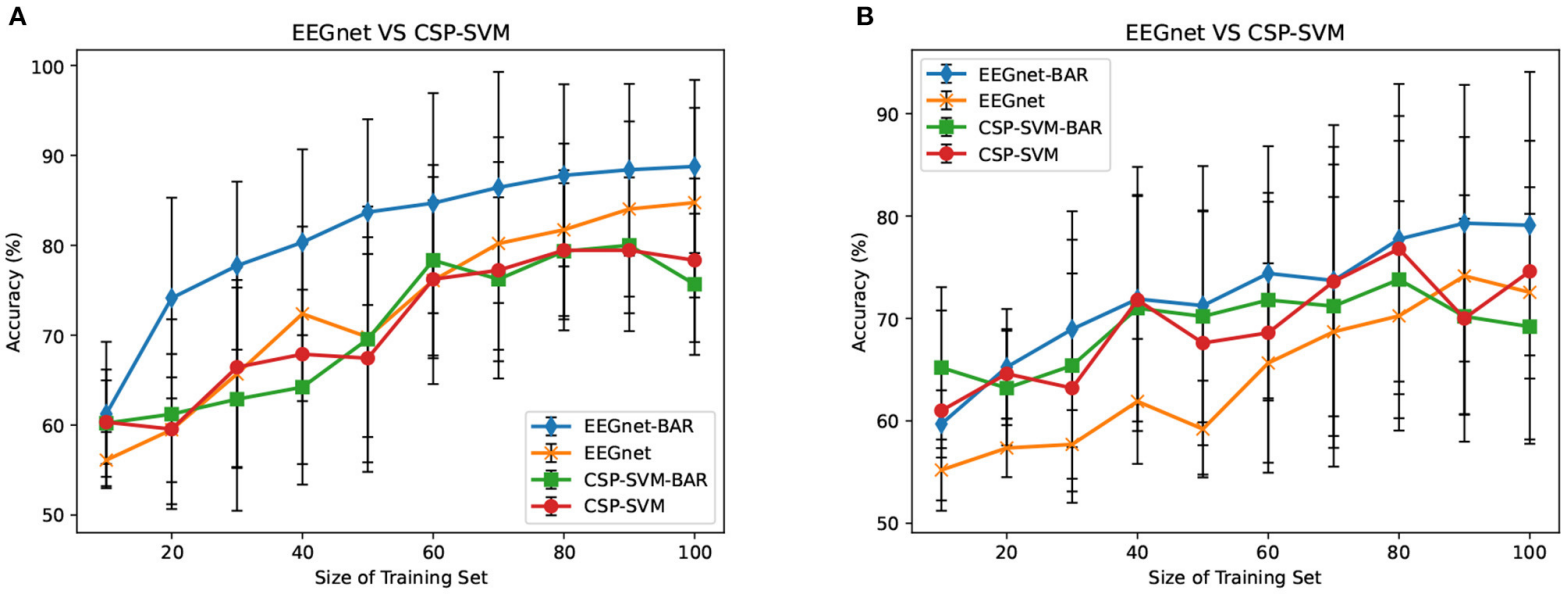
EEGnet vs. CSP-SVM on dataset 1 **(A)** and dataset 2 **(B)**.

The CSP-SVM, a traditional classifier for MI-EEG signals, does not support the AS-schema. Therefore, we can only compare the performance of EEGnet and CSP-SVM in IS-schema. The results were plotted in Figure 6. The training set and testing set were the same as those used by EEGnet. In Figure 6A, our BAR method enabled EEGnet to obtain a huge improvement in classification performance compared to CSP-SVM. In Figure 6B, the improvement of classification performance was not obvious, but it still exceeded the performance of traditional CSP-SVM. This showed that our method can enable deep learning models to be more fully trained, whether the quality of the dataset was poor or better, and the classification performance exceeded the traditional method CSP-SVM's.

### 3.4. Data Visualization

**Figure 7:**
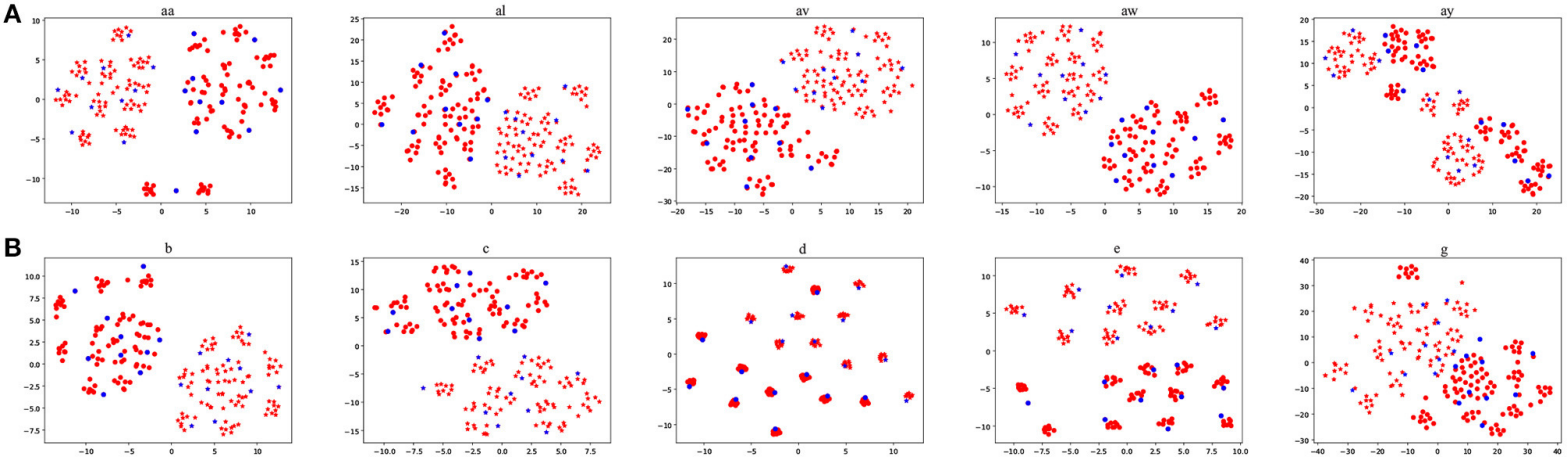
The red points were artificial samples by the proposed BAR and the blue ones were sampled from the recording process. The star points (“*”) and circle points (“o”) represent the first class and the second class samples, respectively.

It is interesting to visualize the locations of the EEG trials generated by our proposed BAR. We used t-Stochastic Neighbor Embedding (t-SNE) (Maaten and Hinton, 2008), a non-linear dimensionality reduction technique that embeds high-dimensional data in a two-or-three-dimensional space, to show and compare the original EEG trials and generated EEG trials in the intra-subject schema. Figure 7 shows the result of t-SNE on augmented training dataset from each subject, where the size of the training set we used is 20. An overall characteristic can be found that BAR's generated EEG trials may not be scattered far away from the original EEG trials. Note that the subject d and subject e are artificially generated “participants.” So that the artificial samples closely surround real samples, which is slightly different from other subjects'.

### 3.5. Experimental Setup

We used ubuntu 18.04.5 LTS with a GPU TITAN V as the experiment platform. We chose 60 epochs determined by our iterated experiments for early stopping. We set the batch size to 16. Adam optimizer was used in all experiments with *lr* = 0.001, β_1_ = 0.9, β_2_ = 0.999.

## 4. Discussion

This article developed a new DA method to generate artificial EEG data from the recorded samples. These artificial data can be used to supplement the training set, which can improve the EEGnet's decoding accuracy. The human brain is composed of two parts, the left hemisphere and the right hemisphere. Many studies have shown cooperative relationships between brain areas under specific tasks (Rubinov and Sporns, 2010). We believe that the motor imagery induced EEG patterns contain three parts: the left hemibrain independent component, the right hemibrain independent component, and the left and right brain cooperative components. Moreover, our data augmentation method may strengthen the left and right brain collaboration components through channel-level reorganization and constructs its more robust training samples. We designed two schemas toward two application scenarios: single-subject scenario and multi-subjects scenario. We have demonstrated that the augmented datasets significantly improved the performance of detection in deep-learning-based MI-BCI systems. Furthermore, we reimplemented the noise-added method and the flipping method, known as common DA methods for time series data (Wen et al., 2020). The results showed that the proposed BAR significantly outperformed them in the MI-EEG classification task. Although the final binary classification accuracy has not improved much, it is a reliable improvement because it passed the *t*-test.

Merely expanding the size of the training dataset can improve the classification performance of the deep learning network. To get a more objective conclusion, the expansion ratio of the noise-added DA method was set to be the same as our proposed BAR's. Moreover, the results were plotted in Figure 4, which illustrated that with the help of our proposed BAR method, the EEGnet had been more fully trained to achieve the best classification performance. We found that as the size of the training set increases, the classification accuracy of deep learning increases very quickly at the beginning. After a certain threshold, the increase rate will slow down. In the Figures 4A.1,A.2 the threshold is 20. In the Figures 4B.1 the threshold is 40. In the Figure 4B.2 the threshold is 70.

The flipping method destroyed the time-domain characteristics of EEG signals. Comparing Figures 4A.1,A.2, we found that the performance of the flipping method had dropped. In these two schemas, the only difference was that EEGnet was pre-trained in the mixed dataset of multiple subjects in the second schema. The spatial distribution characteristics of datasets mixed by multiple subjects would be reduced by the differences between subjects (Ang et al., 2008). Therefore, training EEGnet on a multi-subject mixed data would force the model to pay more attention to temporal features. However, the temporal features had been destroyed by the flipping method's operation in the time axis. EEGnet would perform worse in the AS-schema if the flipping DA method was used. This result was also consistent with prior knowledge that the two important dimensions of motor imaging EEG signal characteristics were space and time (Sakhavi et al., 2018).

The noise-added method was difficult to tune. We implemented two versions for the comparative experiments. One version had the same expansion ratio as the flip method, and the other version had the same expansion ratio as our proposed BAR method. In our experiment, the noise-added method was not adjusted to the optimal state. The tuning process of the noise-added method was complicated and required massive experiments. There were too many factors affecting the noise-added method's performance, such as the type of noise distribution, the signal-to-noise ratio, and the ratio of the generated data volume, original data volume, etc.

The BAR may promote the application of advanced DA methods such as GANs in the BCI field. After long-term development, the Generative Adversarial Network had evolved various variants, improving the training process's stability and the diversity of the generated samples (Goodfellow et al., 2014; Radford et al., 2015; Isola et al., 2017). Nevertheless, its essence was still a deep generative model that contained two deep modules (generator and discriminator), which included massive parameters to be learned (Gui et al., 2020). To obtain a generator with superior performance, a certain amount of data was needed to support generator and discriminator adversarial training. Still, the motivation for data augmentation in the BCI field was that we did not have enough real training data. This was a conflict. Therefore, when the original dataset's size was very small, using advanced methods such as GAN for data augmentation was not a good choice. However, our method was parameterless, and experiments proved that it can still enhance the deep-learning-based classifier under a small training set size. So, we want to say that our method may help GANs to improve their performance. In other words, the BAR may be a parameterless DA method that can assist the parameterized DA method.

Although AS-schema was dependent on the dataset, the DA method we proposed can improve the deep learning model's classification performance. The viewpoint that AS-schema was dependent on the dataset can be understanding by comparing the red line and the orange line in Figures 4A.3,B.3. As can be seen from Figure 4, the data quality of dataset 1 was better than that of dataset 2. The improvement effect of AS-schema on the dataset recorded from the high-quality subjects was more obvious. With the augmentation of our method, the deep learning model can improve the dataset with many poor subjects, which can be seen in Figure 4B.3. On dataset 1 with the better overall quality, our method can further improve the classification performance of deep learning models. The green line beat the others in Figure 4A.3.

DA method was not effective for CSP-SVM (traditional methods), but it was effective for EEGnet (deep learning methods). Two facts may explain the phenomenon. The perspective of features: In the traditional CSP-SVM framework, the features are extracted by the CSP algorithm. Although These features are highly explainable, they are too simple to reflect the data's original appearance. However, the deep-learning-based classifier is an end-to-end method, and the features are automatically learned from the massive training samples. These features, which are learned by many samples, often reflect more information of the original data. The perspective of non-linear fitting ability: The non-linear fitting ability of the SVM comes from the kernel function. Choosing a suitable kernel function is very dependent on experience (Cortes and Vapnik, 1995). However, the non-linear fitting ability of the deep-learning-based classifier is automatically learned from the massive training samples. Many facts have proved that data-driven non-linear expression capabilities are often better than that of manually selected kernel functions in recent years.

An interesting phenomenon discovered by comparing Tables 1, 2 was that the significance of the proposed BAR was reduced in dataset 1. The reason for the result may be that the size of the training set was enough to train a good neural network in the AS-schema. In the pre-training stage, our neural network was first trained on data from other subjects. The amount of data in this stage was large enough to make the neural network to converge to a not bad point. So the effect of our proposed BAR will be reduced in the pre-training schema.

This article has several limitations that call for future investigation. (1) For multi subjects, we use the pre-training pipeline, which is an approach relying on experience stem from natural language processing (NLP) (Xipeng et al., 2020). Experiments show that this pipeline is not completely suitable for the BCI field. It is worth seeking the best way to transfer knowledge from other subjects to the target subject. (2) Influenced by the phenomenon of ERD and ERS, we choose the left brain part and the right brain part as the region to be divided and be recombined. Although we demonstrate this divided approach's effectiveness by extensive experiments, the optimal dividend approach is a crucial problem for the channel-wise-recombined DA method. (3) Through a large number of artificial samples obtained by the BAR in a short time, these samples have a large number of redundant samples. They contain countless repetitive information, which will significantly reduce the training speed of the model. Selecting high-quality samples from artificially generated samples has become a problem, which would become a potential application scenario for active learning (Settles, 2009). These questions will guide our next research direction.

## 5. Conclusion

In this study, a data augmentation method (denoted as BAR) based channel-level recombination was proposed for MI-BCI systems. In our method, to obtain an augmented training set, we divided each sample into two samples according to the brain region to which the channel belongs and then regroup them in the same category. After that, the EEGnet was trained on the augmented training set. Two common DA methods were implemented as comparisons in two training schemas to verify the proposed BAR method. All comparative experimental results passed the paired-sample *t*-test, which fully demonstrated our proposed BAR's effectiveness. At the same time, we found that AS-schema was dependent on the dataset. It performed well on dataset 1 but badly on dataset 2. One possible reason was that dataset 2 was of poor quality, and AS-schema did not apply. How to match the AS-schema with a poor quality dataset will be our next research direction. In bad situations, our method can still improve the decoding performance of deep learning models. The proposed BAR may promote the application of deep learning technology in BCI systems.

## Data Availability Statement

The original contributions presented in the study are included in the article/supplementary material, further inquiries can be directed to the corresponding author/s.

## Ethics Statement

Ethical review and approval was not required for the study on human participants in accordance with the local legislation and institutional requirements. Written informed consent for participation was not required for this study in accordance with the national legislation and the institutional requirements.

## Author Contributions

YP and LX: conceptualization. YP: methodology, data curation, and writing—original draft preparation. HY: software, formal analysis, and visualization. YP, ZL, and YY: validation. YP and JJ: investigation. EY: resources and funding acquisition. YP, ZL, and LX: writing—review and editing. YY and WL: supervision. YP and LX: project administration. All authors: contributed to the article and approved the submitted version.

## Conflict of Interest

The authors declare that the research was conducted in the absence of any commercial or financial relationships that could be construed as a potential conflict of interest.
